# Congestion patterns of electric vehicles with limited battery capacity

**DOI:** 10.1371/journal.pone.0194354

**Published:** 2018-03-15

**Authors:** Wentao Jing, Mohsen Ramezani, Kun An, Inhi Kim

**Affiliations:** 1 Institute of Transport Studies, Department of Civil Engineering, Monash University, Melbourne, Victoria, Australia; 2 The University of Sydney, School of Civil Engineering, Sydney, New South Wales, Australia; Beihang University, CHINA

## Abstract

The path choice behavior of battery electric vehicle (BEV) drivers is influenced by the lack of public charging stations, limited battery capacity, range anxiety and long battery charging time. This paper investigates the congestion/flow pattern captured by stochastic user equilibrium (SUE) traffic assignment problem in transportation networks with BEVs, where the BEV paths are restricted by their battery capacities. The BEV energy consumption is assumed to be a linear function of path length and path travel time, which addresses both path distance limit problem and road congestion effect. A mathematical programming model is proposed for the path-based SUE traffic assignment where the path cost is the sum of the corresponding link costs and a path specific out-of-energy penalty. We then apply the convergent Lagrangian dual method to transform the original problem into a concave maximization problem and develop a customized gradient projection algorithm to solve it. A column generation procedure is incorporated to generate the path set. Finally, two numerical examples are presented to demonstrate the applicability of the proposed model and the solution algorithm.

## Introduction

Battery electric vehicles (BEVs) have received much attention in the past few years due to their advantages in reducing greenhouse gas emissions, noise pollution, reliance on fossil oil and improving the efficiency of electricity grid by vehicle-to-grid technology [1]. Similarly, most of the autonomous vehicles currently under development appear to be electric powered. When they are matured enough to be wide-spread in car manufacturing market, electrified autonomous vehicles are likely to change travel behavior and traffic patterns. Governments and automotive manufacturers have recognized the value of these vehicles in helping the environment and are encouraging BEV ownership through economic incentives and public charging station deployment [2]. Currently, however, BEV users still suffer from the inconvenience of limited driving range, long charging time and insufficient public charging stations [3]. In addition, range anxiety of BEV drivers will inevitably add a certain level of restrictions to BEV drivers’ path choices, at least for a long future period prior to the coverage of recharging infrastructures reaching a sufficient level [4], especially for those BEVs with limited battery capacities.

BEV companies are trying to overcome this limited range requirement by implementing fast charging stations, where a vehicle can be charged in minutes rather than hours to full capacity [5]. However, operating fast charging stations is costly and fast charging reduces the life of a battery due to the irreversible damages to charging cells [6]. Despite the development of fast charging techniques, BEVs still take more time to recharge than the time needed for a standard gasoline vehicle to refuel. Hence, BEV commuters are more likely to charge their vehicles at home rather than at stations [7].

Nevertheless, insufficient charging stations and limited driving range for BEVs make traffic assignment problem (TAP) more challenging due to the incorporation of path distance constraints and battery capacity constraints. The existing TAP models should be modified to better describe commuters’ behavior with the prevalence of BEVs. There have been many endeavors to address this problem. Among which, some studies enforce flow of a path to be zero if the path distance is greater than the driving range limit of BEVs. The classic Frank-Wolfe method with a constrained shortest path algorithm can be applied to solve this problem under deterministic user equilibrium (DUE) [8]. As an extension of static path distance constraint, stochastic range anxiety resulting in stochastic path distance constraint has been considered in networks [9–11]. Network equilibrium problem was further addressed when modeling transportation networks that accommodated both gasoline vehicles (GVs) and BEVs [4, 12, 13]. A multi-class dynamic user equilibrium model was proposed to evaluate the performance of the mixed traffic flow network, where GVs chose paths with minimum travel time and BEVs selected paths to minimize the generalized costs including travel time, energy cost and range anxiety cost. It was also pointed out that the BEV energy consumption rate per unit distance traveled is lower at moderate speed than at higher speed resulting in an equilibrium that BEVs choose paths with lower speed to conserve battery energy [14]. Relay/charging requirement has been taken into account in network equilibrium problems and was formulated as a nonlinear integer programming [15]. It was found that traffic congestion would affect fuel economy of BEVs and BEVs might become more fuel-efficient as the average speed increases, particularly at local arterials [16]. Hence, another work considered recharging time based on flow-independent energy consumption in the base network equilibrium model and further extended the proposed DUE model with flow-dependent energy consumption assumption [3].

However, a more realistic and general situation is that travel time is a random variable and is perceived by travelers in an imperfect, stochastic manner. For example, travel time varies due to stochastic traffic flow conditions. Moreover, battery energy consumption rate is demonstrated to be not only distance-dependent but also time-dependent because it is pointed out that heating and air-conditioning systems of BEVs may consume a substantial amount of energy of the total battery capacity and reduce the BEV’s range limit [17]. Therefore it can be estimated well if link flow volume can be predicted more precisely. Although the stochastic user equilibrium (SUE) principle plays a more realistic role than DUE principle in describing road user’s path choice behavior, the general SUE TAP considering both multinomial logit (MNL) and multinomial probit (MNP) loading with driving range limit constraints has received little attention because of its complexity. As a rational extension of general SUE with flow-independent path distance constraints [18, 19], the SUE traffic assignment model considering flow-dependent link energy consumption is referred to as general SUE traffic assignment with battery capacity constraints.

Previously proposed side constraints for the TAP are basically imposed on traffic flows through nodes, links, paths or O-D pairs and may be grouped into two major categories, i.e. link-based and path-based constraints [9]. The first category is generally referred to as the stochastic TAP with link capacity [see e.g., [20–22]]. A milestone is Meng’s linearly constrained minimization model, which is a general SUE traffic assignment problem with link capacity constraints. This work was inspired by the stochastic social optimum (SSO) traffic assignment with the objective of minimizing the total perceived travel time by Maher, Stewart [23]. Maher, Stewart [23] found that the solution of SSO could be achieved by solving a SUE problem using the marginal cost function. Meng, Lam [22] demonstrated that SUE flow pattern could be generated by solving an SSO problem applying a modified link travel time function. Early developed algorithms to solve the unconstrained logit-based SUE problem were link-based, e.g. [24]. These link-based algorithms do not require path storage and often use Dial’s STOCH algorithm or Bell’s alternative as the stochastic loading step [25, 26].

The second category, the stochastic TAP with path-based constraints, has received much less attention. Only deterministic TAP with flow-independent path-based constraints under BEV scheme has been considered in these studies [3, 4, 8, 12, 15, 19, 27]. In general, they assumed that BEV users could charge only at trip origins and destinations, that the distance of any feasible trip must not exceed the given distance limit [8, 9, 12]. However, for general SUE TAP, no research has taken flow-dependent path energy consumption constraints into the general SUE TAP model.

The main challenge of using a path-based algorithm in the past is the memory requirement. This restriction has been relaxed considerably in recent years due to rapid advances in the computing resources. Different from link-based algorithms above, path-based algorithms require explicit path storage to directly compute the logit path choice probabilities. Olof, Jan [28] developed a path-based algorithm based on the disaggregated simplicial decomposition (DSD) algorithm to solve the MNL SUE problem. Among the path-based algorithms for the traffic equilibrium problem with additive path costs, much attention has been paid to the DSD algorithm and the gradient projection (GP) algorithm [29]. GP algorithm has been shown as a successful path-based algorithm for solving traditional traffic equilibrium problem with additive and non-additive path costs due to its global convergence and simple implementation [30]. A comparison work of evaluating the performance and robustness of these two path-based algorithms can be found in Chen and Lee [31]. Furthermore, to investigate the impact of step size scheme, different step size strategies of the path-based algorithms developed to solve the C-logit SUE models based on an adaptation of the GP method were investigated in [32]. Another inevitable problem of the path-based problem is the way of generating paths. A possible alternative path set can be obtained from a path choice set generation algorithm [33]. Behaviorally, this has an advantage of explicitly identifying paths which are most likely to be used and also allows a greater flexibility to include path-specific attributes that might not be obtainable directly from the link attributes [33, 34].

This paper is concerned with a general SUE TAP with battery capacity constraints, as an extension and generalization of the previous DUE TAP version with driving distance constraints or battery capacity constraints. To the best of our knowledge, it remains to be an open question to find an exact solution method for solving the general SUE problem with path-based constraints, incorporating column generation to avoid path enumeration on a transport network with BEV.

Meng, Lam [22] proposed a solution framework combining Lagrangian dual (LD) method with GP algorithm for general SUE problem with link capacity constraints. However, due to the existence of the implicit path-specific battery out-of-energy function, it remains uninvestigated if this framework can also be applied for path-based constraints. Hence, for the general SUE TAP, we also adopt the solution method framework of combining LD with GP and prove its applicability. Specifically, the path set in this paper is generated prior to the assignment using column generation procedure which has been embedded in the GP algorithm [35] to avoid path enumeration. The GP algorithm iteratively updates the Lagrangian multiplier corresponding to each path, until the optimal solution is obtained.

To sum up, the contributions of this study are threefold. Firstly, to enrich the general SUE family with side constraints (link-based and path-based) and make consistence with side-constrained general DUE condition, it is believed that this is the first paper studying a general SUE model with path-based constraints. Secondly, a holistic methodology is proposed for general SUE traffic assignment model with battery capacity constraints on BEV scheme, in which the path choice is restricted by the battery capacity with a single charge. Thirdly, a Lagrangian dual based exact solution method incorporating column generation is developed for solving this path-constrained general SUE model.

The remainder of this paper is organized as follows. In Sections 2, we elaborate the problem formulation and analyze its solution properties. Section 3 presents an LD reformulation and details its algorithmic implementations by incorporating a convergent GP subroutine and column generation procedure. Section 4 presents the numerical results of applying the algorithm procedure to two case studies. Section 5 provides the concluding remarks.

## Notation, problem description and model formulation

Let us assume the transport network is modeled as a connected graph, denoted by *G* = (*N*,*A*), where *N* and *A* are sets of nodes and links, respectively. (*r*,*s*) stands for certain ordered pairs of nodes, *r* ∈ *R* and *s* ∈ *S*,where node *r* is an origin and node *s* is a destination. *R* ⊂ *N* and *S* ⊂ *N* are sets of origins and destinations, respectively. There are non-negative travel demands *q*^*rs*^ between (*r*,*s*). $q={\left(q_{}^{rs}\right)}^{T},\forall \left(r,s\right)$ is a column vector for all the travel demands. Let *K*_*rs*_ be the set of paths connecting O-D pair (*r*,*s*), $f_{k}^{rs}$ be traffic flow on path *k* ∈ *K*_*rs*_, $f^{rs}={\left(f_{k}^{rs}\right)}^{T},k\in K_{rs}$ be a column vector of all these path flows between O-D pair (*r*,*s*), and $f={\left(f^{rs}\right)}^{T},\forall \left(r,s\right)$ be a column vector of all the path flows over the entire network. Let *v*_*a*_ denote traffic flow on link *a* ∈ *A* and $v={\left(v_{a}\right)}^{T},a\in A$ is a column vector of all link flows. The path flows and link flows should comply with fundamental flow conservation equations:

$$v_{a}=\sum_{\left(r,s\right)}\sum_{k}f_{k}^{rs}\delta_{a,k}^{rs},\forall a\in A$$ (1)

$$q_{}^{rs}=\sum_{k}f_{k}^{rs},\forall (r,s)$$ (2)

$$f_{k}^{rs}\geq 0,\forall (r,s),k\in K_{rs},$$ (3)

where $\delta_{a,k}^{rs}$ = 1 if path *k* ∈ *K*_*rs*_ between O-D pair (*r*,*s*) traverses link *a* ∈ *A*, and 0 otherwise.

Let *t*_*a*_(*v*_*a*_) denote the separable travel time function of link *a* ∈ *A* that is assumed to be a positive, strictly increasing, convex and continuously differentiable function of the traffic flow on the link. All the link travel time functions are grouped into a column vector. $t\left(v\right)={\left(t_{a}\left(v_{a}\right)\right)}^{T},a\in A$. Travel time on path *k* ∈ *K*_*rs*_ between O-D pair (*r*,*s*) can be considered as a function of all the path flows, denoted by $c_{k}^{rs}(f)$ with the expression

$$c_{k}^{rs}(f)=\sum_{a}t_{a}(v_{a})\delta_{a,k}^{rs}$$ (4)

To generate a SUE flow pattern by solving a SSO problem, a modified link travel time function ${\bar{t}}_{a}(v_{a})$ corresponding to link travel time function *t*_*a*_(*v*_*a*_),*a* ∈ *A* is defined to be positive, strictly increasing and continuously differentiable [22].

$${\bar{t}}_{a}(v_{a})=\left\{ \begin{array}{l} \frac{\int_{0}^{v_{a}}t_{a}(x)dx}{v_{a}},v_{a}>0\\ t_{a}(0),v_{a}=0 \end{array}\right.$$ (5)

With modified link travel time functions $\left\{{\bar{t}}_{a}(v_{a}),a\in A\right\}$, the corresponding path modified travel time can be expressed as

$${\bar{c}}_{k}^{rs}(f)=\sum_{a\in A}{\bar{t}}_{a}(v_{a})\delta_{ak}^{rs},\forall (r,s),k\in K_{rs}$$ (6)

Let ${\bar{c}}_{}^{rs}(f)={\left({\bar{c}}_{k}^{rs}(f)\right)}^{T},k\in K_{rs}$ be a column vector of all the modified path travel times between O-D pair (*r*,*s*). In terms of any positive feasible path flow pattern **f**, there are continuously differentiable path-specific dummy functions, ${\bar{d}}^{rs}(f^{rs})\in {ℜ}^{\left|K_{rs}\right|},\forall (r,s)$, so that the conventional SUE conditions associated with the path travel time functions can be satisfied by path flow pattern **f**, $\left\{({\bar{c}}^{rs}(f)+{\bar{d}}^{rs}(f^{rs})\},\forall (r,s\right)$, namely

$$f_{k}^{rs}=q_{}^{rs}\cdot P_{k}^{rs}(({\bar{c}}^{rs}(f)+{\bar{d}}^{rs}(f^{rs}))$$ (7)

where $P_{k}^{rs}(({\bar{c}}^{rs}(f)+{\bar{d}}^{rs}(f^{rs}))$ is referred to as the probability of choosing a given path that has the minimum perceived generalized path cost and $P_{k}^{rs}(({\bar{c}}^{rs}(f)+{\bar{d}}^{rs}(f^{rs}))=\mathrm{Pr}(U_{k}^{rs}<U_{l}^{rs},\forall l\in K_{rs})$. The perceived generalized path cost of any path *k* ∈ *K*_*rs*_ connecting O-D pair (*r*,*s*), $U_{k}^{rs}$ is random variable, where $U_{k}^{rs}={\bar{c}}_{k}^{rs}+{\bar{d}}_{k}^{rs}+\varepsilon_{k}^{rs}$. $\varepsilon_{k}^{rs}$ is the random perception error of the path cost and ${\bar{d}}_{k}^{rs}$ represents an additional additive cost variable across all links associated with path *k* ∈ *K*_*rs*_ to fulfill Eq (7) which is the SUE condition. At the optimum, the additional path-specific cost term ${\bar{d}}_{k}^{rs*}(f^{rs})=\sum_{a}v_{a}(\partial {\bar{t}}_{a}/\partial v_{a})\delta_{a,k}^{rs}$, thus ${\bar{c}}_{k}^{rs}+{\bar{d}}_{k}^{rs}=\sum_{a\in A}({\bar{t}}_{a}+v_{a}(\partial {\bar{t}}_{a}/\partial v_{a}))\delta_{a,k}^{rs}$ representing the induced marginal system cost if a new traveler is added into the system traversing on path *k* ∈ *K*_*rs*_ connecting O-D pair (*r*,*s*) [36] or the so-called marginal social cost [23]. The analytical expressions of ${\bar{d}}^{rs}(f^{rs})$ is presented under logit-based SUE conditions in [23, 36]. The path-specific cost ${\bar{d}}_{k}^{rs}(f^{rs})$ can be expressed as,

$${\bar{d}}_{k}^{rs}=-\frac{1}{\rho }\ln[\frac{f_{k}^{rs}}{q^{rs}}\sum_{k}\exp(-\rho ({\bar{c}}_{k}^{rs}+{\bar{d}}_{k}^{rs}))]-{\bar{c}}_{k}^{rs},\forall (r,s),k\in K_{rs}.$$ (8)

where *ρ* is the scale parameter of the logit model.

To simplify the traffic network modeling, only BEV (as an alternative traffic mode) is considered and a set of assumptions regarding demand heterogeneity and travel behaviors are considered. First, it is assumed that the demand population is only comprised of a single class of BEV. Certainly, if needed, multiple types of BEV with different battery capacities, initial battery charging state (fully charged or not), range anxiety level (a safety margin that BEV drivers would like to reserve before battery depletes) and energy consumption functions can be readily incorporated into the model without changing the problem’s nature and model’s structure [12].

Second, we assume a given fixed travel demand and SUE principle. In other words, stochasticity and elasticity of travel demand are not considered regardless of its stochastic nature [37, 38]. Each BEV traveler chooses a path that minimizes his/her perceived travel cost and no one can reduce his/her perceived cost by unilaterally switching to an alternative path. The travel cost consists of two parts: path energy consumption and possible battery out-of-energy cost. When BEV runs out of battery before reaching destination, battery out-of-energy cost occurs, e.g. a roadside assistance cost. Furthermore, without loss of generality, we assume that BEV travelers use a common form of systematic travel cost for determining their travel choices.

In our network equilibrium analysis, we implicitly assume that all BEV are fully charged at their origins (e.g. home garages), and there is no battery-charging or battery-swapping stations in the network. In most transportation networks, it may take a number of years to deploy sufficient electricity-recharging infrastructures for achieving a certain level of coverage. Consequently, BEV users would choose the path whose energy consumption is less than or equal to the battery capacity, denoted by *D*. Although it is difficult to foresee how future developments in battery and vehicular technologies may enhance the fuel economy of BEVs at various traffic conditions, the link energy consumption in this paper is assumed to increase with the increasing energy consumption of heating and air-conditioning system over time, which is a linear function of link length and modified link travel time, namely, $e_{a}(v_{a})=\alpha l_{a}^{}+\beta {\bar{t}}_{a}(v_{a}),\forall a\in A$ [3]. The authors, however, do not claim the applicability and suitability of the defined energy consumption function for accurate quantification of link energy consumption. One must consider the relationship between energy consumption and travel time (speed). Note that ${\bar{t}}_{a}(v_{a})$ is the modified link travel time function. In practice, each path *k* ∈ *K*_*rs*_ would have a path energy cost and EV drivers have perception error on this cost. Any feasible path flow pattern should satisfy the battery capacity constraints:

$$f_{k}^{rs}(D-\alpha l_{k}^{rs}-\beta {\bar{c}}_{k}^{rs})\geq 0,\forall (r,s),k\in K_{rs}$$ (9)

With the above battery capacity constraints, the generalized path travel cost is defined by

$${\hat{c}}_{k}^{rs}=\alpha l_{k}^{rs}+\beta {\bar{c}}_{k}^{rs}$$ (10)

which means that if the energy consumption is smaller than or equal to the battery capacity, the flow of BEV users going through path *k* is nonnegative,; otherwise, the trip flow should be equal to zero.

$$\left\{ \begin{array}{l} f_{k}^{rs}\geq 0,\mathrm{if}\;\alpha l_{k}^{rs}+\beta {\bar{c}}_{k}^{rs}\leq D\\ f_{k}^{rs}=0,\mathrm{if}\;\alpha l_{k}^{rs}+\beta {\bar{c}}_{k}^{rs}>D \end{array}\right.,\forall (r,s),k\in K_{rs}$$ (11)

**Remark 1**. If we set *α* = 0, the problem would turn into a travel time-constrained SUE TAP; if setting *β* = 0, it becomes a SUE TAP with driving distance constraints. Therefore, flow-dependent battery capacity constraint is a generalization of BEV’s driving distance constraint.

### Model formulation

This section introduces the general SUE traffic assignment model in terms of path flows with battery capacity constraints as follows:

$$\min Z(f)=\sum_{r}\sum_{s}q_{}^{rs}S^{rs}({\bar{c}}_{}^{rs}(f)+{\bar{d}}_{}^{rs}(f^{rs}))-\sum_{r}\sum_{s}\sum_{k}{\bar{d}}_{k}^{rs}(f^{rs})\cdot f_{k}^{rs}$$ (12)

s.t. (1), (2), (3), (9)

where $S^{rs}({\bar{c}}_{}^{rs}(f)+{\bar{d}}_{}^{rs}(f^{rs}))=E[\underset{}{\min}\{{\bar{c}}_{}^{rs}(f)+{\bar{d}}_{}^{rs}(f^{rs})\}]$ is the satisfaction function, i.e., the expected value of the minimum perceived travel time for travelers between O-D pair (*r*,*s*). The satisfaction function is a continuously differentiable, concave function [39]. Compared to the Meng’s model [22] for general SUE with link capacity constraints, the difference lies in the constraints of model (12). This model also possesses two vital propositions as follows.

**Proposition 1**. Any local minimum f* of the minimization model (12)- satisfies the generalized SUE conditions, and the optimal Lagrangian multipliers associated with battery capacity constraint (9) are battery out-of-energy costs.

**Proposition 2**. The SUE link flow pattern induced by any local minimum solution of the minimization model (12)- is unique.

The mathematical proof of proposition 1 can be accessed in the appendix file “**S1 Text**”, while proposition 2 can be proved by following exactly the same procedure as in Meng, Lam [22] and substituting *μ*_*a*_ in their work with *φ*_*a*_ in Eq (17).

### Problem feasibility

The extra battery capacity constraint in the above model could result in problem infeasibility. If the energy consumption of all the paths connecting an O-D pair exceeds the battery capacity, the travel demand between this O-D pair cannot be assigned to the network without causing additional battery out-of-energy cost. The infeasible O-D pairs can be detected by comparing minimum energy cost path with battery capacity under free flow scenarios.

### Solution method

#### Lagrange Dual (LD) method

The objective function (12) includes an inexplicit path-specific dummy functions, ${\bar{d}}^{rs}(f^{rs})$, therefore the original problem cannot be solved directly. Nevertheless, LD formulation of the original model can be established to examine if the proposed algorithms can successfully solve the proposed problem. The solution equivalence between the original problem and the LD problem can be realized if the dual problem is maximized with respect to the Lagrangian multipliers according to the dual theorem.

In order to get the optimal Lagrangian multipliers with respect to the battery capacity constraint in the minimization model, the LD maximization is defined as

$$\underset{\mu \geq 0}{\max}L(\mu )$$ (13)

$$L(\mu )=\underset{f\in \Omega }{\min}[Z(f)+\sum_{r}\sum_{s}\sum_{k}\mu_{k}^{rs}f_{k}^{rs}(\alpha l_{k}^{rs}+\beta {\bar{c}}_{k}^{rs}-D_{})]$$ (14)

where $\mu =(\cdots ,\mu_{k}^{rs},\cdots )\in R^{\left|K_{rs}\right|}$, where $\mu_{k}^{rs}$ is the Lagrangian multiplier associated with battery capacity constraints (9), where |*K*_*rs*_| denotes the number of elements in set *K*_*rs*_. Ω is the set of all the feasible path flows without consideration of battery capacity constraints, i.e. Ω = (**f**|**f** satisfies Eqs (1), (2) and (3)). **μ** acts as a role to convert the battery capacity constraints (9) into the objective function (12). Moreover, *L*(**μ**) is a concave function with respect to non-negative Lagrangian parameter $\mu_{k}^{rs}$.

Following the same procedure in the proof of Proposition 1, it can be demonstrated that any local minimum of above concave function *L*(**μ**) fulfills the conventional SUE conditions (see Eq 6 in Meng, Lam [22]) in terms of the generalized path travel cost function. The well-defined generalized path travel time function is

$${\tilde{c}}_{k}^{rs}=c_{k}^{rs}+\mu_{k}^{rs}(\alpha l_{k}^{rs}+\beta c_{k}^{rs}-D_{}),\forall (r,s),k\in K_{rs}$$ (15)

where $\mu_{k}^{rs}(\alpha l_{k}^{rs}+\beta c_{k}^{rs}-D_{})$ is called the battery out-of-energy cost incurred when the battery energy needed to travel through a given path exceeds the battery capacity of the BEV. The generalized path travel cost and it should satisfy the following conditions:

$$\left\{ \begin{array}{l} \mu_{k}^{rs}=0,\mathrm{if}\;\alpha l_{k}^{rs}+\beta {\bar{c}}_{k}^{rs}\leq D\\ \mu_{k}^{rs}\geq 0,\mathrm{if}\;\alpha l_{k}^{rs}+\beta {\bar{c}}_{k}^{rs}>D \end{array}\right.$$ (16)

Hence, travel time experienced by a driver on a path consists of two parts: normal travel cost and additional cost incurred when energy needed exceeds the battery capacity. The accumulation of the battery out-of-energy cost on a link *a* is defined as:

$$\sum_{r}\sum_{s}\sum_{k}\mu_{k}^{rs}(\alpha l_{k}^{rs}+\beta {\bar{c}}_{k}^{rs}-D_{})\delta_{ak}^{rs}=\sum_{r}\sum_{s}\sum_{k}\lambda_{k}^{rs}\delta_{ak}^{rs}=\sum_{a}\varphi_{a}\delta_{ak}^{rs}$$ (17)

where *φ*_*a*_ accounts all the paths going through it. According to the generalized path travel cost, the generalized SUE conditions that take battery capacity constraints into consideration can be defined as follows.

$$f_{k}^{rs}=q_{}^{rs}\cdot P_{k}^{rs}({\bar{c}}_{}^{rs}(f)+\mu_{k}^{rs}(\alpha l_{k}^{rs}+\beta {\bar{c}}_{k}^{rs}-D_{})),\forall (r,s),k\in K_{rs}$$ (18)

$$f_{k}^{rs}(D_{}-\alpha l_{k}^{rs}-\beta {\bar{c}}_{k}^{rs})\geq 0,\forall (r,s),k\in K_{rs}$$ (19)

$$\mu_{k}^{rs}\geq 0,\forall (r,s),k\in K_{rs}$$ (20)

$$\mu_{k}^{rs}\cdot f_{k}^{rs}(D_{}-\alpha l_{k}^{rs}-\beta {\bar{c}}_{k}^{rs})=0,\forall (r,s),k\in K_{rs}$$ (21)

The generalized link travel time functions can be defined as:

$${\tilde{t}}_{a}(v_{a})={\bar{t}}_{a}(v_{a})+\varphi_{a},a\in A$$ (22)

For any given **μ**≥0, let *v*(**μ**) be the link flow pattern induced from a local minimum of the minimization problem shown in right-hand side of (14). Following the similar proof in Meng, Lam [22], *v*(**μ**) is a unique SUE link flow pattern for networks with the modified path travel time functions and Lagrangian dual formulation (13) is a continuously differentiable concave maximization model. The uniqueness of the optimal link flow solution implies that the gradient of *L*(**μ**) is:

$$\nabla L(\mu )={\left(\cdots ,f_{k}^{rs}(\mu )(\alpha l_{k}^{rs}+\beta {\bar{c}}_{k}^{rs}-D_{}),\cdots \right)}_{\left|K_{rs}\right|}$$ (23)

Applying the Karush-Kuhn-Tucker (KKT) conditions to (13) can lead to proposition 3 that

**Proposition 3.** Assume that μ* is an optimal solution of the LD maximization model (13). *v*(μ*) is the SUE link flow pattern with battery capacity constraint.

Hence, the LD formulation (13) can be efficiently solved by a global convergent GP method with iterative solution updating scheme:

$$\mu^{\left(n+1\right)}=P_{R_{+}^{\left|A\right|}}[\mu^{\left(n\right)}+\alpha_{n}\nabla L(\mu^{\left(n\right)})]$$ (24)

where *n* is the number of iterations; $P_{R_{+}^{\left|A\right|}}[\mu^{\left(n\right)}+\alpha_{n}\nabla L(\mu^{\left(n\right)})]$ is the projection of vector **μ**^(*n*)^ + *α*_*n*_∇*L*(**μ**^(*n*)^) onto the |*A*|-dimensional non-negative orthant, i.e., $R_{+}^{\left|A\right|}$; and the projection operation $P_{R_{+}^{\left|A\right|}}[\cdot ]$ is defined by

$$P_{R_{+}^{}}[y]=\arg\;\underset{x\in R_{+}}{\min}{\sum_{a\in A}\left(x_{a}-y_{a}\right)}^{2}.$$ (25)

Furthermore, {*α*_*n*_} is step size sequence and given at any point **μ**^(*n*)^ ∈ **Q**, where **Q** is the feasible set, denoted by

$$\mu^{\left(n\right)}(\alpha_{n})=P_{Q}[\mu^{\left(n\right)}+\alpha_{n}\nabla L(\mu^{\left(n\right)})],\;\alpha_{n}\geq 0$$ (26)

The unique projection of the vector [**μ**^(*n*)^ + *α*_*n*_∇*L*(**μ**^(*n*)^)] on **Q** where *α*_*n*_ ≥ 0 is a nonnegative scalar parameter. Since the feasible set of **μ** is the whole nonnegative orthant, the Lagrangian multiplier updating formula shown in (24) can be rewritten in the following way:

$$\mu_{l}^{ij}{}^{\left(n+1\right)}=\max\{0,\mu_{l}^{ij}{}^{\left(n\right)}+\alpha_{n}f_{k}^{rs}(\mu^{\left(n\right)})(\alpha l_{k}^{rs}+\beta {\bar{c}}_{k}^{rs}-D_{})\},\forall (i,j),l\in K_{ij}$$ (27)

It has been proved that without the requirement of the Lipschitz condition, every limit point of the sequence {**μ**^(*n*)^} generated by the GP algorithm is a stationary point, as well as a solution point. For step size, a predetermined step size which has simple structure and is commonly used by [Meng and Liu [20], Meng, Lam [22]] is applied in this model instead of the generalized Armijo rule, which belongs to the inexact line search strategies and is for constrained minimization problems. The reason lies in that at each step, the gradient information of the objective function and objective function evaluations are required to determine an appropriate step size to improve the solution when using Armijo rule.

Proposition 3 confirms that solving the SUE link flow pattern with battery capacity constraint can be obtained by solving LD maximization model (13). Although the LD function *L*(**μ**) does not possess an explicit expression, its gradient for any **μ**≥0 can be evaluated by implementing a conventional SUE traffic assignment procedure without consideration of battery capacity constraint. Difficulties in calculating the LD function value and applying Armijo rule render us to employ a GP method with a predetermined step size sequence for solving the continuously differentiable maximization problem (13), which is stated as follows.

**Stage 0: Feasibility Check.** For each O-D pair, find the minimum energy consumption path according to link length and free flow travel time. If the path energy consumed is greater than the BEV battery capacity and the corresponding travel demand is positive, then there is no feasible path between this OD pair without causing additional energy out-of-battery cost. Record this OD pair and infeasible vehicle type to Set A. If Set A is empty, go to the next step; if not, stop.

**Stage 1. Initialization.** Set *v*_*a*_(0) = 0, ${\bar{t}}_{a}=t_{a}[v_{a}(0)]$, iteration counter *n* = 1 and define the path set *K*_*rs*_ = ∅

1 Solve the acyclic K shortest path problem in terms of path energy cost by Yen’s algorithm [40] to generate an initial path set ${\bar{k}}_{rs}(n),K_{rs}={\bar{k}}_{rs}(n)\cup K_{rs}$ and initialize its corresponding multiplier $\mu_{k}^{rs(1)}=0,\forall k\in K$
2 Perform stochastic network loading to assign the travel demand to the paths generated based on ${\bar{t}}_{a}(0)$, $\sum_{}f_{{\bar{k}}_{rs}(1)}^{rs}(1)=q_{rs}$. Logit loading results in K paths while probit loading generates one shortest path between each O-D pair.
3 Assign path flows to links $v_{a}(n)=\sum_{rs}\sum_{k\in K_{rs}}f_{k}^{rs}(n)\delta_{ak}^{rs}$

**Stage 2. Column Generation.** Increment iteration counter *n* = *n*+1

4 Update link travel time ${\bar{t}}_{a}(n)=[v_{a}(n-1)]$ based on ${\bar{t}}_{a}(v_{a})=\left\{ \begin{array}{l} \frac{\int_{0}^{v_{a}}t_{a}(x)dx}{v_{a}},v_{a}>0\\ t_{a}(0),v_{a}=0 \end{array}\right.$ and link energy consumption $e_{a}(n)=\alpha l_{a}^{}+\beta {\bar{t}}_{a}(n-1),\forall a$
5 Solve the K minimum path energy cost problem to generate new paths ${\bar{k}}_{rs}(n)$ and initialize the corresponding Lagrangian multiplier $\mu_{k}^{rs(n)}$ of the newly generated paths.
8.1 Update path set $K_{rs}(n)={\bar{k}}_{rs}(n)\cup K_{rs}(n-1)$, if ${\bar{k}}_{rs}(n)\notin K_{rs}(n-1)$; otherwise use current path set *K*_*rs*_(*n*) in stochastic network loading procedure.

**Stage 3. Equilibration.** Compute the generalized path travel cost $C_{k}^{rs(n)}={\bar{c}}_{k}^{rs}+\mu_{k}^{rs}(\beta {\bar{c}}_{k}^{rs}+\alpha l_{k}^{rs}-D)$ ∀*k* ∈ *K*_*rs*_(*n*)

6 Perform stochastic network loading procedure (logit or probit) to generate new path flow patterns $f_{k}^{rs}(\mu^{\left(n\right)})$ in terms of the current path set *K*_*rs*_(*n*)
7 Obtain the set of link flows according to link/path incidence relationship $V_{a}(\mu^{\left(n\right)})=\sum_{r}\sum_{s}\sum_{k}f_{k}^{rs}\delta_{a,k}^{rs},\forall a\in A$
8 Average flow. Let *v*_*a*_(**μ**^(*n*)^) = [(*n*−1)*v*_*a*_(**μ**^(*n*−1)^)+*V*_*a*_(**μ**^(*n*)^)]/*n*, ∀*a* ∈ *A*
9 Check stopping criterions of both flow change rate and Lagrangian multiplier change rate. (Flow change rate can be referred to page 301 for probit loading and page 327 for logit loading respectively in Sheffi [39].) If the following criterion hold, then terminate.
  $$\underset{}{\max}\{\left|\mu_{k}^{rs(n)}-\max[0,\mu_{k}^{rs}{}^{\left(n\right)}+\alpha_{n}f_{k}^{rs}(\mu^{\left(n\right)})(\alpha l_{k}^{rs}+\beta c_{k}^{rs}-D_{})]\right|\}\leq \varepsilon ,\;\forall \mu_{k}^{rs}$$
10 Otherwise, update Lagrangian multipliers according to the following equation:

$$\mu_{k}^{rs(n+1)}=\max\{0,\mu_{k}^{rs(n)}+\alpha_{n}f_{k}^{rs}(\mu_{k}^{rs(n)})(\alpha l_{k}^{rs}+\beta c_{k}^{rs}-D_{})\}$$

Note that {*α*_*n*_} is a predetermined step size sequen ce satisfying the three conditions:

$$0<\alpha_{n}<1\;\mathrm{and}\;\underset{n\to \infty }{\lim}\alpha_{n}=0;\sum_{n=1}^{\infty }\alpha_{n}=+\infty ;\sum_{n=1}^{\infty }\alpha_{n}^{2}<\infty$$

## Numerical examples

This section presents 2 numerical case studies to assess the performance and properties of the proposed method.

**Fig 1:**
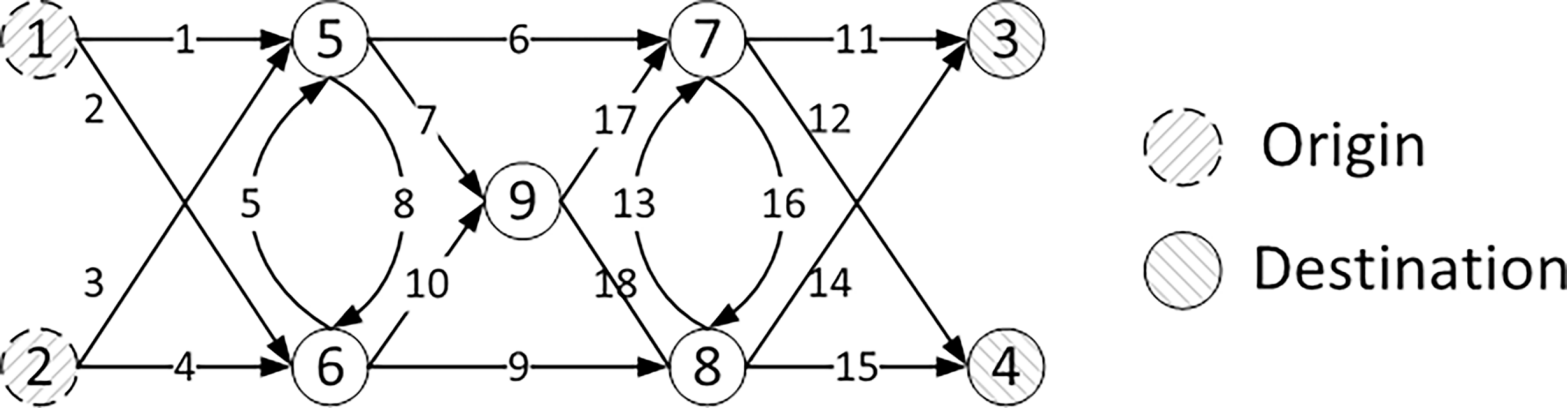
Small network schematic with 2 origins, 2 destinations, 9 nodes, and 18 links.

The first example, which is also adopted by Nie, Zhang [41] and Meng and Liu [20], consists of 9 nodes, 18 links, and 4 O-D pairs: (1,3), (1,4), (2,3), and (2,4), as shown in **Fig 1**. The free-flow travel time is used as a proxy for the link length for each link. Travel time on each link is defined by the following BPR (Bureau of Public Road) type function

$$t_{a}\left(v_{a}\right)=t_{a}^{0}\left(1+0.15\times {\left(\frac{v_{a}}{H_{a}}\right)}^{4}\right),a\in A$$ (28)

where $t_{a}^{0}$ is the free flow travel time and *H*_*a*_ is link *a* capacity. OD demands, free-flow travel time and link capacity are the same as that in Meng, Lam [22].

**Fig 2:**
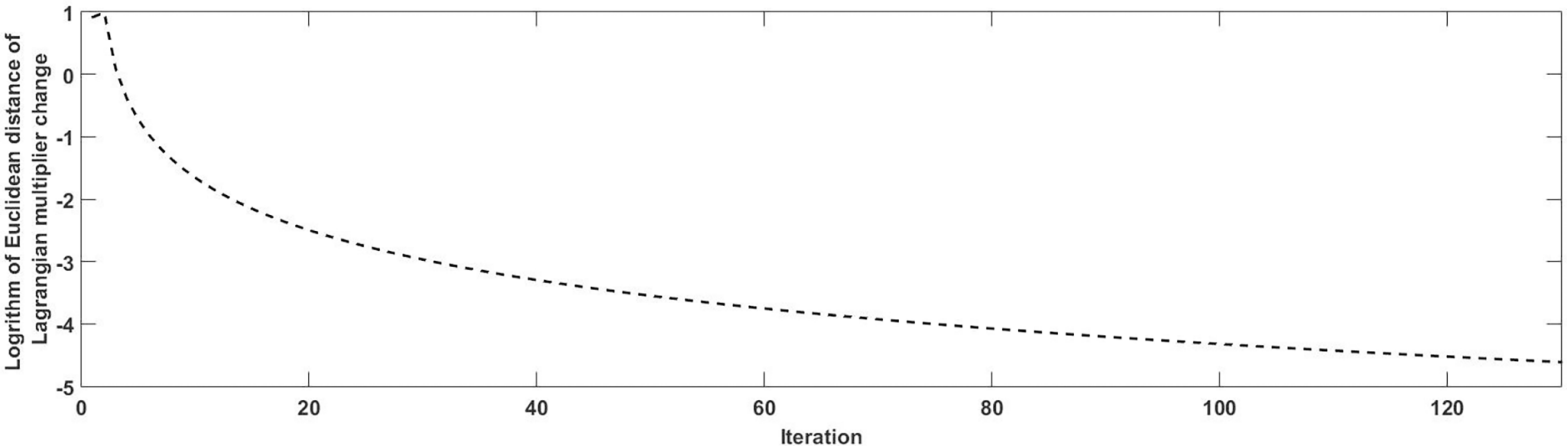
Convergence performance of MNL loading for the small-sized case study.

We use this example to evaluate the performance of proposed algorithms for solving both logit-based and probit-based SUE TAP with battery capacity constraints (further details are provided in **S1 Matlab Code**). The generated paths and their corresponding Lagrangian multipliers at the equilibrium under MNL are shown in **Table 1**. EV range limit is set to 4, and K, α, β are 6, 0.174 and 0.116 respectively. The convergence criterion of both flow change rate and Lagrangian multiplier change rate is 0.01. The step size sequence {*α*_*n*_} and the initial multiplier $\mu_{k}^{\left(0\right)}$ are 1/*n* and 0, respectively. The non-zero multipliers indicate that the energy consumptions of traveling on these paths exceed the BEV battery capacity at the equilibrium, while zero multipliers (e.g. for paths 1-5-7-3 and 1-6-5-7-3) denote paths within the battery capacity, which will not trigger the out-of-energy cost. The number of paths generated in the column generation procedure is related to the value of K. **Fig 2**shows the convergence performance of the solution method under MNL loading, where the equilibrium is reached after 130 iterations. Note that, in **Fig 2**y-axis is in logarithm unit. The Euclidean distance equals to $\underset{\mu }{\max}\left|\mu_{k}^{rs(n+1)}-\mu_{k}^{rs(n)}\right|$.

**Table 1:** Path sets and corresponding optimal Lagrangian multipliers for MNL.

O-D pair	Path generated and its Lagrangian multiplier						
(1,3)	[1,5,7,3] **0**	[1,6,5,7,3] **0**	[1,5,7,8,3] **0.63**	[1,6,8,7,3] **4.23**	[1,5,9,7,3] **5.24**	[1,6,9,7,3] **5.24 **	[1,6,8,3] **4.61**
(1,4)	[1,5,7,4] **0**	[1,5,7,8,4] **0**	[1,6,8,4] **7.07**	[1,6,5,7,4] **8.34**	[1,6,5,7,8,4] **10.83**	[1,6,8,7,4] **10.79**	[] **/**
(2,3)	[2,5,7,3] **0**	[2,5,7,8,3] **0**	[2,6,5,7,3] **10**	[2,5,9,7,3] **9.95**	[2,6,8,7,3] **12.92**	[2,6,8,3] **12.45**	[] **/**
(2,4)	[2,5,7,4] **0**	[2,5,7,8,4] **0**	[2,6,8,4] **18.54**	[2,5,9,7,4] **17.17**	[2,6,5,7,4] **17.13**	[2,5,9,7,8,4] **14.79**	[] /

Furthermore, we perform a thorough sensitivity analysis with respect to travel demand, battery capacity and the logit parameter. The high demand is double of the medium demand in the first numerical example. **Table 2**demonstrates that after a certain level of battery capacity (e.g. battery capacity equals to 6 and 7), there is no influence on the equilibrium link flows, whereas in extreme cases (e.g. battery capacity = 2) where battery capacity is too small to travel through any path between an O-D pair, every corresponding Lagrangian multiplier would be positive and each EV user would experience the battery out-of-energy cost. In addition, higher travel demand may impose congestion on the network and thereby increase the energy consumption rate for the same path comparing to the original demand because of the increasing path travel time. **Table 2**shows that the equilibrium link flow pattern is affected by travel demand and battery capacity. For example, link flows of 9, 11, 12, and 13 in the fifth and eighth column have much difference with each other because the network becomes congested and link travel time goes up when travel demand is high and increasing path travel time results in more energy consumption and more paths infeasible on which BEV will run out of energy and incorporate additional out-of-battery cost.

**Table 2:** Equilibrium link flow for different scenarios of the travel demand and battery capacity under MNL network loading.

Link No.	Link capacity	Medium demand				High demand	
		capacity = 2	capacity = 4	capacity = 6	capacity = 7	capacity = 4	capacity = 6
1	40	21.36	23.85	14.46	14.06	14.38	20.39
2	30	8.64	6.15	15.54	15.94	45.62	39.61
3	50	68.44	68.31	52.20	47.71	39.22	66.20
4	80	1.56	1.69	17.80	22.29	100.78	73.80
5	30	0.00	0.00	0.00	4.40	14.14	2.97
6	60	92.62	94.58	71.22	61.67	21.55	81.34
7	30	3.00	1.47	11.27	14.35	40.18	6.47
8	30	5.81	3.89	15.83	18.65	22.27	4.18
9	90	2.46	3.53	16.47	19.38	92.92	102.82
10	30	1.92	0.42	1.04	4.60	45.36	9.37
11	30	39.03	35.65	30.11	27.71	35.82	10.39
12	30	60.00	37.49	31.70	30.17	2.21	0.00
13	30	0.62	24.43	26.81	29.32	44.48	83.50
14	30	0.97	4.35	9.89	12.29	44.18	69.61
15	30	0.00	22.51	28.30	29.83	117.79	120.00
16	30	2.11	1.10	5.08	6.58	11.47	1.14
17	40	4.92	1.89	12.31	18.95	49.49	11.40
18	30	0.00	0.00	0.00	0.00	36.04	4.44

**Table 3**shows path usage status, revealing the number of total generated paths and the proportion of feasible paths without additional out-of-battery cost corresponding to the scenarios in **Table 2**. When the battery capacity is extremely small and travel demand is medium, e.g. medium demand, capacity = 2, all the generated paths exceed range limit and every path user would experience a battery out-of-energy cost. However, while the battery capacity increase to 4, comparing two scenarios of different demand, all paths in the highly congested network are still out of range limit, because the energy consumption increase sharply as the path travel time increases. For the medium demand case, there are at least 2 paths within the range limit for each O-D pair.

**Table 3:** Path status under different travel demand and battery capacity for MNL.

O-D pair	The number of paths within range limit V.S. total paths generated					
	Medium demand				High demand	
	capacity = 2	capacity = 4	capacity = 6	capacity = 7	capacity = 4	capacity = 6
(1,3)	0/7	1/7	7/7	8/8	0/19	3/12
(1,4)	0/10	0/6	5/6	8/8	0/24	3/16
(2,3)	0/6	1/6	4/6	7/10	0/21	2/15
(2,4)	0/10	1/6	2/6	7/8	0/24	1/16

**Fig 3:**
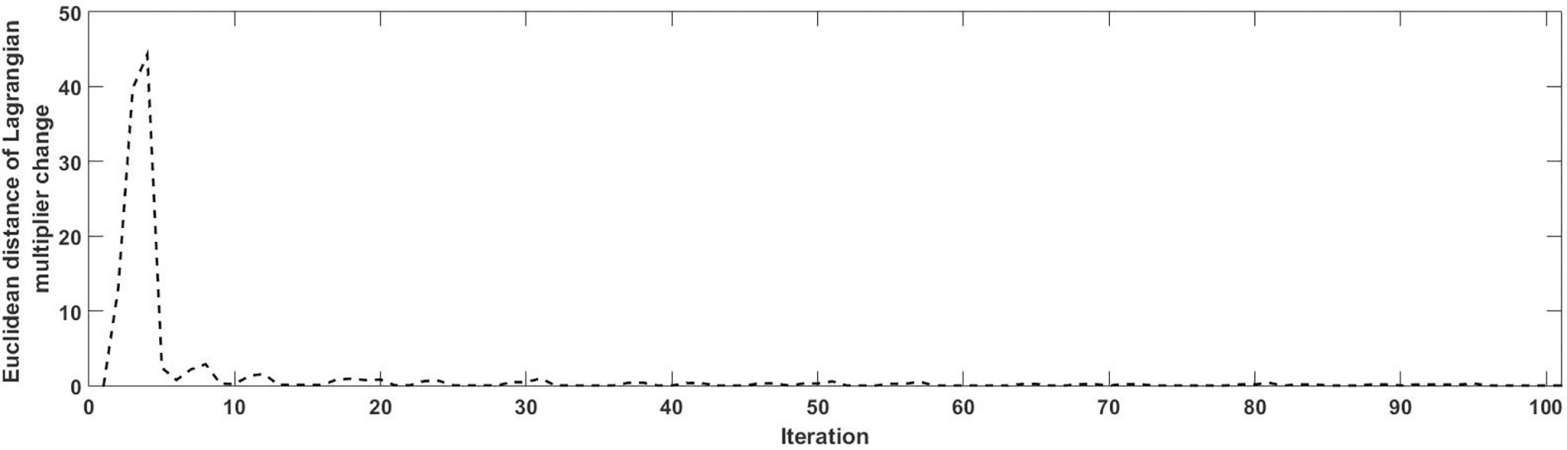
Convergence performance under MNP loading for the small-sized case study.

For MNP, K is set to be 1 because the all-or-nothing assignment is applied in MNP loading and only the shortest path is used to load the demand. Sample size of drawing perceived travel time in Monte Carlo simulation is 200. **Fig 3**shows the convergence performance under MNP loading where there is a fast trend during the first 10 iterations while equilibrium is reached at iteration 100. The equilibrium path sets and their corresponding Lagrangian multipliers are listed in **Table 4**. It is observed that the number of paths generated is affected by travel demand as well. Comparing to MNL, fewer paths are used between each O-D pair because the all-or-nothing assignment is used in probit loading step to assign the travel demand to the shortest path. When the demand is low and the network is not congested, only several paths would be calculated in column generation step as the shortest path are stored in path sets. A sensitivity analysis is conducted with respect to probit parameter in **Table 5**. As we can see, the effect of changing probit parameter values is not that obvious in terms of the link flow volume. In MNL, different K values in K-shortest path algorithm used for column generation step would lead to different path size. It is well known that MNL model suffers from independence of irrelevant alternative (IIA) property [39], which is the reason why larger K value is used in MNL model.

**Table 4:** Path sets for MNP and its Lagrangian multiplier.

O-D pair	Medium demand		High demand		
(1,3)	[1,5,7,3] **0**	[] **/**	[1,5,7,3] **0**	[1,6,8,3] **0**	[] **/**
(1,4)	[1,5,7,4] **0**	[1,5,7,8,4] **0.35**	[1,5,7,4] **0**	[1,6,8,4] **0**	[] /
(2,3)	[2,5,7,3] **0**	[] **0**	[2,5,7,3] **0**	[2,6,8,3] **0**	[] /
(2,4)	[2,5,7,4] **0**	[2,5,7,8,4] **0.26**	[2,5,7,4] **0**	[2,6,8,4] **0**	[2,5,7,8,4] **2.63**

**Table 5:** Equilibrium link flow for different scenarios of the travel demand and probit parameter under MNP network loading.

Link No.	Medium demand		High demand	
	Parameter = 0.2	Parameter = 1.2	Parameter = 0.2	Parameter = 1.2
1	30.00	29.80	29.84	30.53
2	0.00	0.20	30.16	29.47
3	70.00	70.00	111.77	108.60
4	0.00	0.00	28.23	31.40
5	0.00	0.00	0.00	0.00
6	100.00	99.80	141.61	139.12
7	0.00	0.00	0.00	0.00
8	0.00	0.00	0.00	0.00
9	0.00	0.20	58.39	60.88
10	0.00	0.00	0.00	0.00
11	40.00	40.00	72.58	71.05
12	45.74	49.11	61.94	63.16
13	14.26	10.69	7.10	4.91
14	0.00	0.00	7.42	8.95
15	14.26	10.89	58.06	56.84
16	0.00	0.00	0.00	0.00
17	0.00	0.00	0.00	0.00
18	0.00	0.00	0.00	0.00

**Fig 4:**
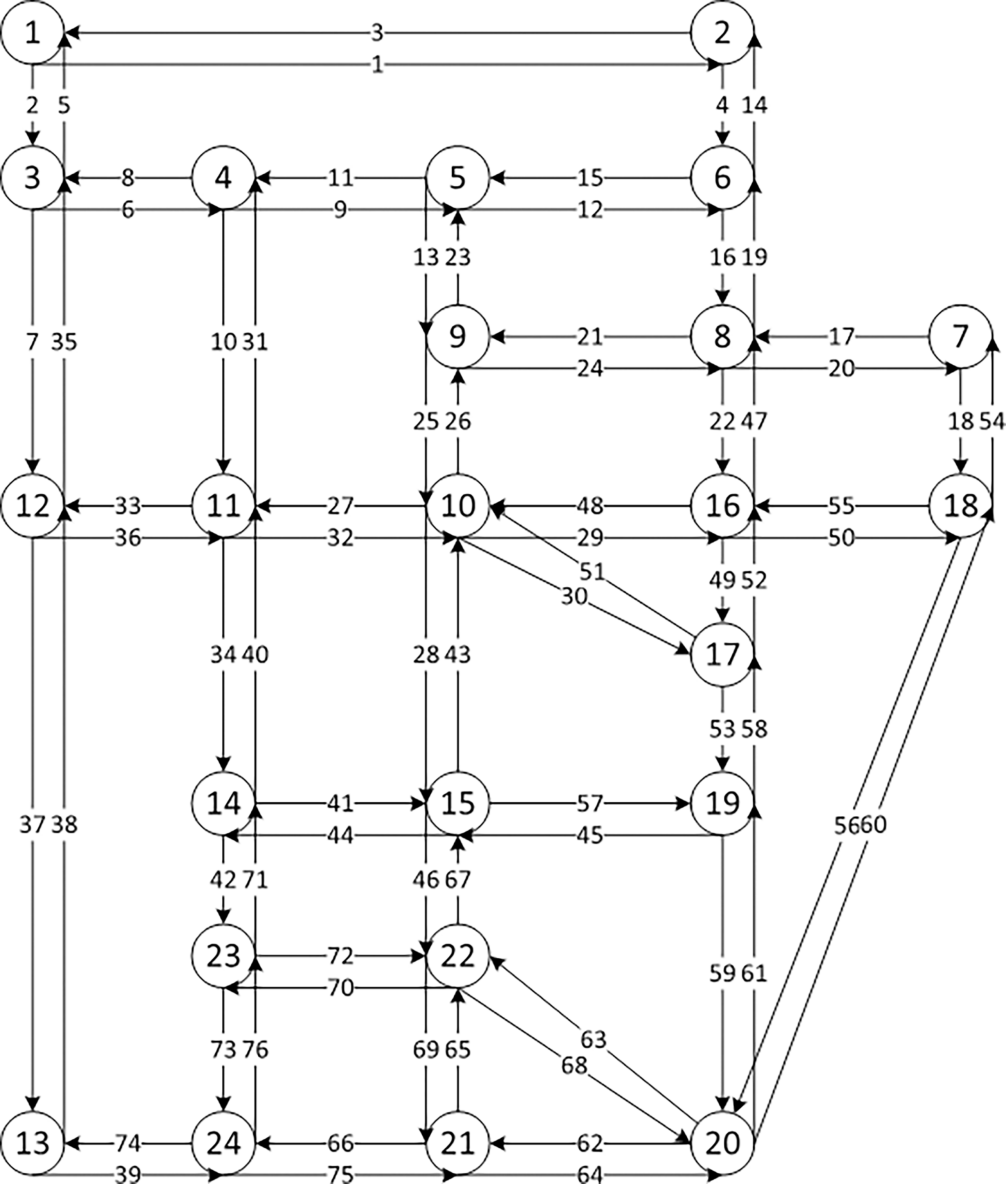
Sioux falls network with 24 nodes and 76 links.

For the second case study, a variation of the Sioux Falls network (see Fig 4) is adopted which has been chosen as a benchmark network in numerous traffic assignment studies [Suwansirikul, Friesz [42]]. One particular reason for presenting the Sioux Falls network example here is to highlight the effect of parameter setting on computational cost. This network consists of 24 nodes, 76 links, and 576 O-D pairs. For computational experiments, the number of iterations (ITR) and the total computational cost (TCC) were compared for MNL and MNP under different battery capacities (BC), stochastic parameter values, and K values. The weight value of link energy consumption function, namely α, β, and convergence criteria used here are the same as the first example.

**Table 6 and Table 7**list the computational cost with different parameters under logit and probit-based loading. Assuming the travel cost coefficient of the logit model, referred to as logit parameter, is 0.2, it can be seen from **Table 6**that K value has a great impact on computational cost. By looking into ITR before convergence and comparing the first two scenarios, bigger K value would decrease the ITR needed while increasing the TCC. Clearly, most computational cost is spent on calculating the K shortest paths at column generation and logit loading steps for each iteration. Therefore, a more efficient K-shortest path algorithm would improve TCC. According to these two tables, it can also be observed that smaller battery capacity, bigger stochastic parameters and larger battery capacity, lead to slow convergence speed. Intuitively, the larger the BC is, the more paths can be selected in the path set. More time is needed to generate the paths, calculate the path choice probability, and assign the flows. When BC is large enough and travel demand is fixed, BEV can actually travel to every destination with no concern about running out of energy, thus making it a conventional SUE with no additional battery capacity constraints. In reality, BEV may not fully charged under some circumstances, e.g. power grid failure, multiple trips. Therefore, multi-class users with different battery capacities can be further taken into consideration without changing the problem’s structure.

**Table 6:** Computational cost with different parameter settings for MNL.

	K = 3,logit parameter = 0.2				K = 6,logit parameter = 0.2			
BC	0.05	0.2	0.6	1	0.05	0.2	0.6	1
ITR	36	26	8	4	28	21	11	6
TCC(s)	136.21	102.06	27.98	12.62	285.61	209.10	106.75	54.21
	K = 6,logit parameter = 0.4				K = 6,logit parameter = 1			
BC	0.05	0.2	0.6	1	0.05	0.2	0.6	1
ITR	20	13	5	3	12	6	3	2
TCC(s)	204.58	131.88	44.81	23.10	124.51	56.61	23.09	13.59

**Table 7:** Computational cost with different parameter settings for MNP.

	K = 1,probit parameter = 0.2				K = 1,probit parameter = 0.4			
BC	0.05	0.2	0.6	1	0.05	0.2	0.6	1
ITR	8	6	6	3	6	6	6	3
TTC(s)	6.40	5.07	5.21	2.63	4.89	4.90	4.63	2.55
	K = 1,probit parameter = 1				K = 1,probit parameter = 2			
BC	0.05	0.2	0.6	1	0.05	0.2	0.6	1
ITR	6	3	3	3	6	3	3	3
TTC(s)	4.91	3.12	2.67	2.60	4.55	2.86	2.46	2.56

Moreover, the bigger value of the stochastic parameter, the larger is the random perception error on both travel time and energy cost. From the results, it is found that it took less time and less iterations for probit-based network loading to converge than that of logit-based network loading. This result is because the all-or-nothing assignment is used in probit-based loading. Only the shortest path is generated between each O-D pair at each iteration. When BC is relatively large, all the paths energy consumption would be within the capacity level and Lagrangian multipliers are equal to zero.

## Conclusions

This paper works on the stochastic traffic assignment models with battery capacity constraints, where new path-constrained stochastic user equilibrium (SUE) traffic assignment problem is formulated, solved and numerically analyzed. The method considers a flow-depend energy consumption assumption for battery electric vehicles (BEV), which is a generalization of flow-independent driving distance constraint. The BEV’s range limit is determined based on both its travel distance and travel time that is a function of traffic congestion. Flow-dependent constraint inevitably calls for fundamental changes to the existing network flow modeling tools for properly capturing traffic patterns and evaluating traffic assignment results. It is proved that the solution method framework, LD-GP-stochastic network loading, could be applied not only in link-based problems but also in path-based problems. In this path-based SUE problem, the column generation procedure is applied to the path choice set generation which turns out to works well with GP and stochastic network loading and provides basic insights of solving path-constrained SUE problem to avoid path enumeration. The application of the algorithms in the small network justifies the applicability of the solution procedures to general network with path-based constraints. The numerical analysis results show the impact of battery capacity, travel demand and stochastic parameters on network equilibrium flow and computational cost.

As a pure mathematical modeling tool to characterize BEVs’ travel behavior in the network with some ideal socioeconomic assumptions, we expect that the modeling technique and solution methods demonstrated in this work would potentially trigger the interest of investigating other types of stochastic traffic assignment problems with path-based constraints in logit-type or weibit route choice models. The model itself can also be applied for more accurate quantification of network flows, travel demand and battery capacity levels. As a modeling platform for more practical and realistic model, the proposed model should be enhanced to accommodate mixed traffic flows of different types of vehicles such as BEVs, hybrid vehicles and conventional gasoline vehicles as well as the availability of charging infrastructure. Our future study will investigate the possibility of incorporating charging time, range anxiety level and value of time in model extensions. Based on the SUE models proposed in this paper, we will also investigate how to optimally locate charging stations in the network in terms of different objectives.

## Supporting information

S1 Matlab Code Details of the numerical test of the first example including code and network attributes. (ZIP)

S1 Text Proof of Proposition 1. (DOCX)
