# Supplementary figures and images for: Congestion patterns of electric vehicles with limited battery capacity
Source: PLoS One. 2018 Mar 15;13(3):e0194354. doi: 10.1371/journal.pone.0194354 (PMC5854388; doi:10.1371/journal.pone.0194354)

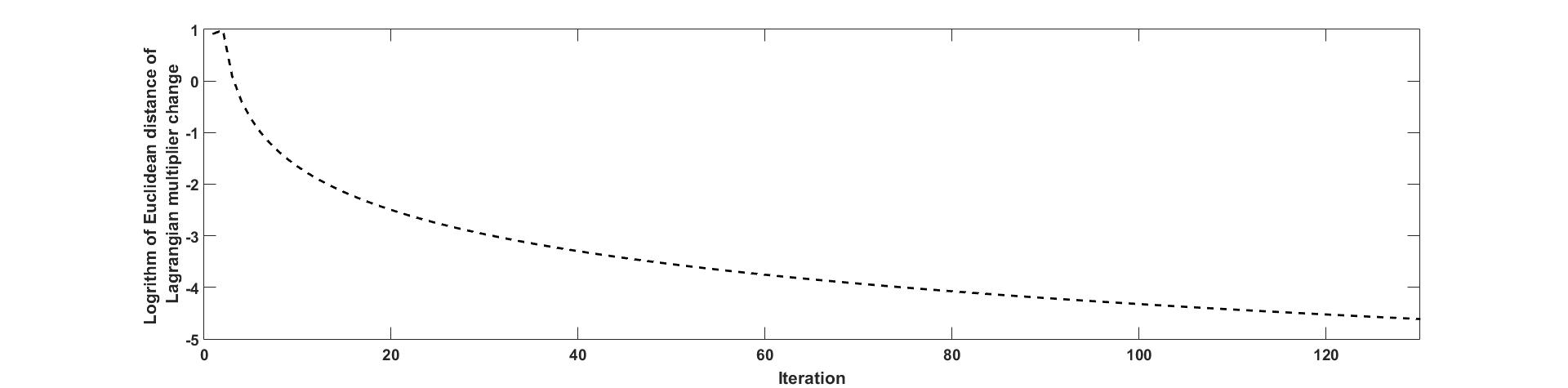

Supplement: S1 Matlab Code — (ZIP) [file pone.0194354.s001.zip › MNL (Small network)/MNL.jpg]
