# Supplementary material for: Congestion patterns of electric vehicles with limited battery capacity
Source: PLoS One. 2018 Mar 15;13(3):e0194354. doi: 10.1371/journal.pone.0194354 (PMC5854388; doi:10.1371/journal.pone.0194354)
Supplement: S1 Text — (DOCX) [file pone.0194354.s002.docx]

**S1 Text. Proof of Proposition 1**

Consider the Lagrangian function of the minimization model with constraints below.

$$\begin{matrix} L(\mathbf{f},\mathbf{\mu})=\sum_{r} \sum_{s} q^{rs}S^{rs}(\overline{\mathbf{c}}^{\mathbf{rs}}\mathbf{(f)}+\overline{\mathbf{d}}^{\mathbf{rs}}\mathbf{(f}^{\mathbf{rs}}\mathbf{)})-\sum_{r} \sum_{s} \sum_{k} \overline{d}_{k}^{rs}(f^{rs})\cdot f_{k}^{rs}-\sum_{r} \sum_{s} \sum_{k} \mu_{k}^{rs}f_{k}^{rs}\cdot(D-\alpha l_{k}^{rs}-\beta\overline{c}_{k}^{rs}) \\ +\sum_{r} \sum_{s} \pi^{rs}(q^{rs}-\sum_{k} f_{k}^{rs}) \end{matrix}$$

where $\mathbf{\mu}$ is the Lagrangian multiplier with respect to the battery capacity constraints, and row vector $\mathbf{\mu}=(...,\mu_{k}^{rs},...)\in R^{\left|K_{rs}\right|}$.

Keep in mind that the vector $\overline{\mathbf{d}}^{\mathbf{rs}}\mathbf{(f}^{\mathbf{rs}}\mathbf{)}$ is a function of path flows between O-D pair $(r,s)$ only. Take partial derivative of Lagrangian function above with respect to path flow $f_{l}^{ij}$on a designated path $l\in K_{ij}$ between O-D pair $(i,j)$ can yield

$$\begin{matrix} \frac{\partial L(\mathbf{f},\mathbf{\mu})}{\partial f_{l}^{ij}}=\sum_{r} \sum_{s} q^{rs}(\sum_{k\in K_{rs}} P_{k}^{rs}\cdot\frac{\partial\overline{c}_{k}^{rs}(\mathbf{f})}{\partial f_{l}^{ij}})+q^{ij}\sum_{k\in K_{ij}} (P_{k}^{ij}\cdot\frac{\partial\overline{d}_{k}^{ij}(\mathbf{f}^{\mathbf{ij}})}{\partial f_{l}^{ij}})-\overline{d}_{l}^{ij}(\mathbf{f}^{\mathbf{ij}})- \\ \sum_{k\in K_{ij}} f_{l}^{ij}\frac{\partial\overline{d}_{k}^{ij}(\mathbf{f}^{\mathbf{rs}})}{\partial f_{l}^{ij}}+[\mu_{l}^{ij}\cdot(\alpha l_{l}^{ij}+\beta\overline{c}_{l}^{ij}-D)+\sum_{r} \sum_{s} \sum_{k\in K_{rs}} \mu_{k}^{rs}f_{k}^{rs}\beta\frac{\partial\overline{c}_{k}^{rs}(\mathbf{f}^{\mathbf{rs}})}{\partial f_{l}^{ij}}]-\pi^{ij} \end{matrix}$$

The first term of right-hand side (RHS) of the equation above can be rewritten by

$$\begin{matrix} \sum_{r} \sum_{s} q^{rs}(\sum_{k\in K_{rs}} P_{k}^{rs}\cdot\frac{\partial\overline{c}_{k}^{rs}(\mathbf{f})}{\partial f_{l}^{ij}})=\sum_{r} \sum_{s} q^{rs}\sum_{k\in K_{rs}} P_{k}^{rs}\sum_{a\in A} \frac{d\overline{t}_{a}(v_{a})}{dv_{a}}\cdot\delta_{ak}^{rs}\cdot\delta_{al}^{ij} \\ =\sum_{r} \sum_{s} f_{k}^{rs}\sum_{k\in K_{rs}} \delta_{ak}^{rs}\sum_{a\in A} \frac{d\overline{t}_{a}(v_{a})}{dv_{a}}\cdot\delta_{al}^{ij}=\sum_{a\in A} v_{a}\cdot\frac{d\overline{t}_{a}(v_{a})}{dv_{a}}\cdot\delta_{al}^{ij} \\ \end{matrix}$$

The second and fourth terms can be canceled out. The fifth term equals to

$$\mu_{l}^{ij}\cdot(\alpha l_{k}^{rs}+\beta\overline{c}_{k}^{rs}-D)+\sum_{r} \sum_{s} \sum_{k\in K_{rs}} \mu_{k}^{rs}f_{k}^{rs}\beta\frac{\partial\overline{c}_{k}^{rs}(\mathbf{f}^{\mathbf{rs}})}{\partial f_{l}^{ij}}=\mu_{l}^{ij}\cdot(\alpha l_{l}^{ij}+\beta\overline{c}_{l}^{ij}-D)+\beta\mu_{l}^{ij}\sum_{a} v_{a}\frac{d\mathop{t_{a}}\limits^{-} (v_{a})}{dv_{a}}\delta_{al}^{ij}$$

Hence, the equation can be simplified as

$$RHS=(1+\beta\mu_{l}^{ij})c_{l}^{ij}-\overline{c}_{l}^{ij}-\mu_{l}^{ij}\cdot(D-\alpha l_{k}^{rs})-\overline{d}_{l}^{ij}(\mathbf{f}^{ij})$$

Since$f^{*}$ is a local minimum, according to KKT conditions, there are optimal Lagrangian multiplier $\mu^{*}$ such that

$$\frac{\partial L(\mathbf{f}^{\mathbf{*}},\mathbf{\mu}^{\mathbf{*}})}{\partial f_{l}^{ij}}=0,l\in K_{ij},i\in R,j\in S$$

$$f_{l}^{*ij}(D-l_{l}^{ij})\geq0,\forall i,j,l$$

$$\mu_{l}^{*ij}\geq0,\forall i,j,l$$

$$\mu_{l}^{*ij}\cdot f_{l}^{*ij}(D-l_{l}^{ij})=0,\forall i,j,l$$

$$\overline{d}_{l}^{ij}(\mathbf{f}^{\mathbf{*rs}})=(1+\beta\mu_{l}^{*ij})c_{l}^{*ij}-\overline{c}_{k}^{*rs}-\mu_{l}^{*ij}\cdot(D-\alpha l_{k}^{rs})$$

$$\begin{matrix} \overline{d}^{ij}(\mathbf{f}^{\mathbf{*rs}})=\sum_{l\in K_{ij}} \sum_{m} \overline{d}_{lm}^{ij}(\mathbf{f}^{\mathbf{*rs}})=\sum_{l\in K_{ij}} [(1+\beta\mu_{l}^{*ij})c_{l}^{*ij}-\overline{c}_{l}^{*ij}-\mu_{l}^{*ij}\cdot(D-\alpha l_{l}^{ij})] \\ =c^{*ij}-\overline{c}^{*ij}+\sum_{l\in K_{ij}} \mu_{l}^{*ij}(\beta c_{l}^{*ij}+\alpha l_{l}^{ij}-D) \\ \end{matrix}$$

Let $\lambda_{l}^{*ij}=\mu_{l}^{*ij}(\beta c_{l}^{*ij}+\alpha l_{l}^{ij}-D)$, $\mathbf{\lambda}^{\mathbf{*ij}}=(...,\lambda_{l}^{*ij},...)\in R^{\left|K_{ij}\right|}$

Thus

$$\overline{d}^{ij}(\mathbf{f}^{\mathbf{*rs}})=c^{*ij}-\overline{c}^{*ij}+\mathbf{\lambda}^{\mathbf{*ij}}$$

where $\lambda_{l}^{*ij}=\mu_{l}^{*ij}(\beta c_{l}^{*ij}+\alpha l_{l}^{ij}-D)$ is the battery out-of-energy cost incurred when the energy needed to travel through a given path exceeds the battery capacity of the EV. The Lagrangian multiplier $\mu_{l}^{*ij}$ stands for an equivalent travel cost value of the unit energy.

$$f_{l}^{*ij}=q^{ij}\cdot P_{l}^{ij}(c^{ij}(\mathbf{f}^{\mathbf{*}})+\mathbf{\lambda}^{\mathbf{*ij}}),\forall(i,j),l\in K_{ij}$$

Eq. , - state that $\mathbf{f}^{\mathbf{*}}$ fulfills the generalized SUE conditions and that $\{\mu_{k}^{rs}\}$ is the relevant SUE battery out-of-energy cost pattern.
